# Advanced Sensors for Real-Time Monitoring Applications ‖

**DOI:** 10.3390/s26092703

**Published:** 2026-04-27

**Authors:** Olga Korostynska, Alex Mason

**Affiliations:** 1 Department of Mechanical, Electronic and Chemical Engineering (MEK), Faculty of Technology, Art and Design (TKD), Oslo Metropolitan University (OsloMet), 0166 Oslo, Norway; 2 Faculty of Science and Technology (REALTEK), Norwegian University of Life Sciences (NMBU), 1432 Ås, Norway; alex.mason@nmbu.no

**Keywords:** healthcare, biopotential, heart rate, PPG, residual cancer burden, motion capture, photoacoustic spectroscopy, intelligent transportation systems, biodegradable implants, remote monitoring

## Abstract

In the world of electronics, sensors are more than just components—they are the eyes, ears, and touchpoints of modern technology. From self-driving cars that rely on LiDAR and ultrasonic sensors to navigate complex environments, smart watches that detect your every move and heartbeat, to advanced brain chip implants that can sense your thoughts and translate them into physical moves with the assistance of exoskeletons, sensors bridge the gap between the physical world and digital systems. The rapid arrival of advanced Artificial Intelligence (AI) and Large Language Models (LLMs) has transformed almost every part of technology, especially data processing. However, the development of sensors remains a vitally important topic. Sensors form the foundation of innovation in electronics; novel sensors provide reliable data across a broad range of application areas and are a foundation for intelligent systems. Notably, knowing the capabilities and limitations of each sensor type is crucial for selecting the right sensor for a specific application, troubleshooting issues, and optimizing system performance. This book, entitled “Advanced Sensors for Real-Time Monitoring Applications II”, demonstrates developments of real sensors for a range of applications, including descriptions of fundamental principles of operation, concepts, theory, and practical validation of the results, as well as a review of current state-of-the-art and future directions.

## 1. Introduction

The human body is a remarkable example of how sensory schemes seamlessly integrate sensory information with biomechanical and physiological processes to enable effective functioning [1]. Sensors enable us to perceive the world, interact meaningfully, and make informed decisions based on our experiences [2]. Thus, sensory information is not merely a passive data stream, but a dynamic and multifaceted landscape that holds invaluable clues about our health and well-being [3]. Sensory schemes can also be harnessed to personalize rehabilitation therapies [4,5], optimize athletic performance [6], and enhance human–computer interactions. Sensor research is paving the way for a future where personalized healthcare is not a distant dream but a tangible reality, empowered by sensors and electronics [7].

Healthcare and related applications take the central place in this book, with a comprehensive review on biopotential signal monitoring systems in a rehabilitation setting, setting the tone in Contribution 1. It is followed by articles focusing on heart rate estimation (Contribution 2), residual cancer burden (Contribution 3), cognitive load with heart rate variability monitoring (Contribution 4), remote responders bio-signal monitoring (Contribution 5), biodegradable implants status (Contribution 6); plantar pressure monitoring (Contribution 7), assembly posture recognition (Contribution 8) and activity monitoring (Contributions 9 and 10).

Another set of articles reports on advanced sensors for real-time monitoring applications of gas sensing (Contributions 11 and 12), intelligent transportation systems (Contribution 13), water velocity (Contribution 14), and temperature monitoring (Contribution 15). A more detailed overview of each contribution is given in the next section.

## 2. Overview of Contributions

A timely review of biopotential signal monitoring systems in rehabilitation is offered in Contribution 1. This paper provides a state-of-the-art review of the recent advances in electroencephalogram (EEG), electrocardiogram (ECG), and electromyogram (EMG) signal monitoring systems and sensors, both commercial and research, that are relevant to the field of tele-rehabilitation and health monitoring. Future advancements in sensor technology must focus on developing low-cost, miniaturized, comfortable, and clinically relevant wearable devices designed for real-life conditions to bridge this gap and support widespread home-based and remote healthcare applications.

Precision heart rate estimation using a PPG sensor patch was achieved in Contribution 2. With the help of new algorithms of pre-quality checking and Hankel decomposition, motion artifacts due to subjects’ hand motions and walking were considerably reduced. The proposed method achieved the best accuracy of 3.78 bpm in mean absolute errors among all previously reported studies for hand-moving scenarios.

Residual cancer burden is a prognostic tool widely used to estimate survival outcomes in breast cancer, and a novel optical sensory system for its assessment is reported in Contribution 3. It is based on machine learning of data acquired from 15 patients undergoing neoadjuvant chemotherapy and has achieved an accuracy of 0.98 in predicting RCB number/class based on the changes in optical properties measured by the Opti-scan probe. These findings suggest that the reported approach has considerable potential as a valuable non-invasive tool for monitoring breast cancer patients’ response to NAC and guiding treatment decisions.

Cardiac surgery is one of the most complex and high-risk areas of medicine. Effective team coordination requires the management of interruptions and distractions as well as awareness of the context of the surgical procedure. Cognitive load, a measure of how mentally occupied or stressed a team member is at a particular time, provides a useful metric for understanding how team members’ attention and mental load vary through the stages of a surgical procedure. An open-source, interoperable architecture for generating real-time surgical team cognitive alerts from heart-rate variability monitoring was the scope of the work reported in Contribution 4. This novel work demonstrates for the first time the feasibility of acquiring multiple ECG waveforms, applying an interoperable pipeline capable of filtering the ECGs, identifying IBIs, calculating HRV metrics, estimating team cognitive load states, and generating alerts in response. For example, if the ECG is sampled at 100 Hz, each sample covers a 10 ms time period, and then the errors become similar in magnitude to the beat-to-beat variability being measured. This prototype application requires a sample rate of 500 measurements per second or higher to reduce the magnitude of timing measurement errors.

Remote responder bio-signal monitoring in a cooperative human–robot architecture for search and rescue (SAR) in disaster scenarios was investigated in Contribution 5. The roles of emergency responders are challenging and often physically demanding, so it is essential that their duties are performed safely and effectively. The integration of a set of health monitoring sensors suitable for detecting stress, anxiety, and physical fatigue in an Internet of Cooperative Agents architecture for search and rescue missions was proposed, which allows remote control and communication between human and robotic agents and the mission control center. Proof-of-concept experiments were performed with a bio-signal sensor suit worn by firefighters in two high-fidelity SAR exercises. ECG, EDA, PZT, and EEG data, a key combination of bio-signals for physical fatigue, anxiety, and stress detection, were monitored. The proposed designs were assessed by a Fire Brigade Consortium in two workshops in a 90,000 m^2^ area plot, divided into several zones. Notably, the gain of EEG sensors (41,782) was much higher than the gains of the other sensors (e.g., 1100 for ECG, and 1 for PZT), so EEG is more sensitive to electromagnetic noise and may not be reliable in some environments.

Biodegradable materials such as magnesium (Mg) and its alloys have increasingly been used in orthopedic surgery for bone fixation. The use of near-infrared spectroscopy for the in vivo monitoring of such biodegradable implants is reported in Contribution 6. It is a non-destructive, non-invasive technique used to monitor tissue oxygenation in various clinical settings. NIR light is estimated to reach a 3–3.5 mm depth, and the diffuse light is detected by the optical fiber inside the probe. The purpose of this study was to investigate the effects on hemoglobin changes and tissue oxygen saturation (StO_2_) after the implantation of Mg-alloy (WE43) and titanium (Ti) implants using a multiwavelength optical probe. Interestingly, the StO_2_ values of days 0 and 45 for the Mg-implanted animals were similar, but StO_2_ mainly changed postoperatively from day 0 to day 3, possibly due to bubble gas formation, a well-known side effect associated with the initial fast degradation of Mg implants. It was shown that applying the modified Beer–Lambert law (MBLL) to particular measurement conditions related to the effect of Mg degradation within tissues can enhance the reliability of data on the target tissue’s physiological state and biochemical content.

The study of human walking, namely gait analysis, can be used as a valuable diagnostic tool to distinguish between normal and pathological gait. A novel 3D-printed capacitive smart insole for plantar pressure monitoring was presented in Contribution 7. The sensor exhibited a sensitivity of 1.19 MPa^−1^, a wide working pressure range (<872.4 kPa), excellent stability and durability (at least 2.280 cycles), great linearity (*R*^2^ = 0.993), fast response/recovery time (142–160 ms), low hysteresis (*DH* < 10%) and the ability to support a broad spectrum of gait speeds (30–70 steps/min). Authors state that depending on the 3D printing systems, one can have a pair of smart insoles ready within a day, which makes them perfect for personalized applications.

A novel, cost-effective method for recognizing assembly postures in Industry 4.0 settings using Inertial Measurement Units (IMUs) is presented in Contribution 8. It proposes a low-cost human motion collection system, combining BMI160 IMU modules with ESP8266 WiFi for real-time data transmission. This data is then used to calculate human posture by mapping IMU trackers to a digital human skeleton in Unity, enabling synchronized 3D visualization, with a reported average accuracy of 98.24%. The study validates this approach through experiments simulating assembly tasks in a virtual reality environment, collecting motion data from six key joints.

Activity monitoring based on the structural vibration of ambient objects was the focus of the research reported in Contribution 9. Here, a vibration sensing device using a six-axis IMU and an optimized beam structure achieved a 6.2-times-higher vibration amplitude and an increase in signal energy of 480% when compared to a directly mounted IMU without a beam. The sensor fusion algorithm provided a noise reduction of 5.6% by fusing accelerometer and gyroscope data at 103 Hz.

Another physical activity monitoring paper by the same authors also focused on the non-contact method using a multi-axial IMU in animal husbandry, Contribution 10. The activity was classified as resting, stationary activity, and locomotion with over 90% accuracy.

Detection of SO_2_F_2_ using a photoacoustic two-chamber approach was the focus of the study reported in Contribution 11. SO_2_F_2_ is highly toxic to humans and the environment, but is widely used for termite control in buildings, warehouses, and shipping containers. Thus, reliable real-time detection is necessary, and the novel system for that was developed based on the photoacoustic detection method. The results show that concentrations below 1 ppm SO_2_F_2_ can be reliably detected.

Drone-mountable gas sensing platform using graphene chemiresistors for remote in-field monitoring was the focus of the research reported in Contribution 12. An array of graphene-based field-effect transistors in combination with commercial humidity and temperature sensors is used to relay data on the presence of airborne chemicals over distances of 1 km by wireless communication. A system on a drone was controlled by a mobile application for real-time data transmission, compatible with Internet of Things (IoT) applications. The information collected by all sensors comprised in the platform was registered every 210 ms (at a rate of 4.8 Hz). However, the device performs a moving average to reduce noise, yielding a time resolution of ~1 s and matching the time resolution of the BME280 sensor.

The increasing attacks on traffic signals worldwide indicate the importance of intrusion detection, and this issue was researched in Contribution 13, where an intelligent transportation system was proposed with a technique to detect such intrusions. Specifically, it is based on the real-time observation of traffic parameters, namely, traffic flow, average vehicle speed, phase time of the signal, and their historical values for Melbourne Central Business District, combined with an approach based on Shannon’s entropy to incorporate the uncertainty of observed data. An overall accuracy of 79.3% for intruded traffic conditions was demonstrated.

Monitoring flow velocity in storm and wastewater systems is crucial for environmental applications, including pollution load estimation and normalization of pathogen concentrations in wastewater. The work in Contribution 14 presents a low-cost, low-power water velocity sensor utilizing acoustic Doppler measurement. This sensor is significantly cheaper than the typical commercial alternatives and is very energy-efficient: 2 mW for 10 measurements an hour. Its compact size also supports the deployment of the sensor for real-time monitoring in large quantities.

A fiber optic sensing system consisting of a fiber Bragg grating (FBG) sensor, optical circulator, optical band pass filter, and photodetector was developed to monitor the real-time temperature response of a structure under a dynamic thermal loading in Contribution 15. This system showed a fast response during 40 °C/min thermal loading and is suitable for online structural health monitoring.

## 3. Conclusions

Despite the unprecedented rise in AI data processing algorithms in the last year, real-time sensors remain the indispensable source of information for countless applications, forming the bedrock upon which intelligent systems are built. This book details developments in healthcare, activity and posture monitoring, gas detection, environmental, traffic, and infrastructure monitoring, and search and rescue operations. Advanced sensors for real-time monitoring applications will continue to play a pivotal role in the future, enabling smarter decision-making, enhanced safety, and greater efficiency across all areas.
